# Info-gap management of public health Policy for TB with HIV-prevalence and epidemiological uncertainty

**DOI:** 10.1186/1471-2458-12-1091

**Published:** 2012-12-19

**Authors:** Yakov Ben-Haim, Clifford C Dacso, Nicola M Zetola

**Affiliations:** 1 Yitzhak Moda’i Chair in Technology and Economics, Technion—Israel Institute of Technology, Haifa 32000, Israel; 2 Molecular & Cell Biology and Medicine, Baylor College of Medicine, Houston, Texas; 3 Division of Infectious Diseases, Department of Medicine, University of Pennsylvania, Philadelphia, Pennsylvania

**Keywords:** TB management, HIV-AIDS, Public health, Epidemiology, Uncertainty, Robustness, Info-gap

## Abstract

**Background:**

Formulation and evaluation of public health policy commonly employs science-based mathematical models. For instance, epidemiological dynamics of TB is dominated, in general, by flow between actively and latently infected populations. Thus modelling is central in planning public health intervention. However, models are highly uncertain because they are based on observations that are geographically and temporally distinct from the population to which they are applied.

**Aims:**

We aim to demonstrate the advantages of info-gap theory, a non-probabilistic approach to severe uncertainty when worst cases cannot be reliably identified and probability distributions are unreliable or unavailable. Info-gap is applied here to mathematical modelling of epidemics and analysis of public health decision-making.

**Methods:**

Applying info-gap robustness analysis to tuberculosis/HIV (TB/HIV) epidemics, we illustrate the critical role of incorporating uncertainty in formulating recommendations for interventions. Robustness is assessed as the magnitude of uncertainty that can be tolerated by a given intervention. We illustrate the methodology by exploring interventions that alter the rates of diagnosis, cure, relapse and HIV infection.

**Results:**

We demonstrate several policy implications. Equivalence among alternative rates of diagnosis and relapse are identified. The impact of initial TB and HIV prevalence on the robustness to uncertainty is quantified. In some configurations, increased aggressiveness of intervention improves the predicted outcome but also reduces the robustness to uncertainty. Similarly, predicted outcomes may be better at larger target times, but may also be more vulnerable to model error.

**Conclusions:**

The info-gap framework is useful for managing model uncertainty and is attractive when uncertainties on model parameters are extreme. When a public health model underlies guidelines, info-gap decision theory provides valuable insight into the confidence of achieving agreed-upon goals.

## Background

Public health policies affect millions of people and determine the allocation of health care funds. However, selecting an intervention for a given population at a given time is highly uncertain. Data supporting public health decisions are scarce, of poor quality, not fully generalizable and lack appropriate controls [1]. The high uncertainty in infectious disease epidemiology results also from inter-dependency among individuals. When prospective studies or randomized controlled trials are available, they usually represent selected groups with as little variance as possible and may not apply to other populations [2]. Such lack of generalizability may be more problematic for the recommendations developed by international organizations. Those guidelines use the best available information and expert opinion. Nonetheless the yield, effectiveness and cost of the interventions vary significantly due to heterogeneity of the populations in which they are implemented [1,3].

Science-based mathematical models commonly support public health decisions [4-7]. Many models were developed to explain or predict the course of an epidemic for specific interventions. However, these models are limited by the uncertainty of the data and assumptions they employ [5,7].

Despite severe uncertainty in public health decision-making, actions must be timely and cost-effective. Analysis of uncertainty is central in responsible decision making using uncertain data and models.

Information-gap (info-gap) theory [8] was developed for decision making when knowledge gaps are substantial, worst cases cannot be reliably identified, and probability distributions are unreliable or unavailable. An info-gap model is the disparity between what *is known* and what *needs to be known* in order to achieve an acceptable outcome. The focus is on robustly achieving satisfactory outcomes, thus making this technique suitable for public health policy decision making [9]. Info-gap theory has been applied in engineering, biological conservation, economics, project management, medicine and homeland security (see http://info-gap.com).

We develop a framework for the practical use of info-gap theory in public health for controlling infectious diseases. We focus on tuberculosis (TB) in the context of pandemic HIV as an example.

## Methods

### Epidemiological background

The World Health Organization reported 9.4 million incident TB cases and 1.7 million TB deaths in 2009 and estimated that only 63% of annual incident TB cases were detected and reported; of these, 86% were successfully treated [10,11]. Given the disease burden, the United Nations Millennium Development Goals include targets and indicators related to TB control. The targets include decreasing TB incidence by 2015, halving TB prevalence and mortality by 2015 (compared with 1990), and diagnosing 70% of new smear-positive cases and curing 85% of these cases by 2015. However, despite current efforts, many countries will not achieve these targets [10-14].

The HIV-AIDS pandemic is the major worldwide challenge to TB control [11,13,15,16]. HIV creates a situation of serious uncertainty for public health interventions based on pre-HIV era models [10,11,13]. This is reflected in population distribution, spread, control, and recurrence. Latently and actively infected individuals contribute differently to spread of disease. It is necessary to consider infectivity, rapidity of progression, re-infection, individuals with higher susceptibility for infection and reinfection resulting from HIV coinfection, etc. in order to produce refined models of diagnosis and treatment.

Many different epidemiological models have been used to evaluate treatment strategies. Deterministic compartment models are the most common, and we use a slightly modified version of the widely used Murray-Salomon model [17-19] to describe the evolution of TB/HIV epidemics under various scenarios. The details of the model appear in Appendix “The Murray-Salomon model” section.

### Info-gap theory

The robustness function is the basic decision-support tool in an info-gap analysis. If our dynamic model were accurate we could evaluate any proposed intervention in terms of the outcome of that intervention that is predicted by the model. An intervention with low predicted TB prevalence is preferred over an intervention with higher predicted prevalence.

The problem is great model uncertainty. This means that predicted outcomes are unreliable and it is unrealistic to prioritize interventions in terms of their predicted outcomes. Using the model to find the intervention whose predicted outcome is best, is not suited to planning with highly uncertain models.

Model-based predictions are useful, but when deciding which public health intervention to implement, we should also ask: how wrong could the model be, and an acceptable outcome is still guaranteed? For any specified intervention we ask: what is the largest error in the model, up to which all realizations of the model would yield acceptable outcomes? Equivalently, what outcomes can reliably be anticipated from this intervention, given the unknown disparity between the model and reality? Answers to these questions lie in the robustness function, specified in Appendix “Definition of robustness” section. The robustness is dimensionless, and equals the greatest fractional error in the model parameters that is consistent with a specified outcome requirement. We use the robustness function to prioritize the interventions in terms of their robustness against uncertainty for achieving the required outcome.

Knight [20] recognized that probability distributions are sometimes unknown and that severe uncertainty may be non-probabilistic. Wald [21], Ben-Tal and Nemirovski [22] and others developed tools for robustly managing non-probabilistic uncertainty by minimizing the worst outcome on a set of possibilities. Info-gap theory is non-probabilistic and handles situations where worst cases are unknown.

We summarize here the main attributes of the info-gap robustness function: a plot of robustness-to-uncertainty versus required performance. This is the basic info-gap tool for prioritizing available options.

#### Robustness trades off against performance [23,24]

More demanding performance requirements are less robust against uncertainty than less demanding requirements. This trade off is quantified and expressed graphically by the monotonic robustness curve.

#### Model predictions have zero robustness against uncertainty [25]

When models are highly uncertain, it is unrealistic to prioritize one’s options based on predicted outcomes of those options, because those predictions have no robustness to errors in the underlying models. Options must be evaluated in terms of the level of performance that can be reliably achieved; this is expressed by robustness.

Combining the trade off and zeroing properties yields realistic prioritization of options.

#### Prioritization of options depends on performance requirements

Prioritization of options may change as requirements change. This is called “preference reversal” and is expressed by the intersection of the robustness curves of different options. Preference reversal provides insight to anomalous behavior such as the Ellsberg and Allais paradoxes in human decision making [8], the equity premium puzzle in economics [8], and animal foraging [26]. We will show that preference reversal occurs when selecting public health interventions because priorities are time- and context-dependent.

#### Info-gap models of uncertainty are non-probabilistic

Info-gap robustness analysis is implementable even when probability distributions are unknown, and thus is suited to severe uncertainty. In contrast, Monte Carlo simulation, Bayesian analysis, or probabilistic risk assessment require knowledge of probabilities. Other non-probabilistic tools include interval analysis, fuzzy set theory [27], possibility theory [28] and Robust Decision Making (RDM). A comparison of info-gap and RDM has recently been published [29].

#### Info-gap is operationally distinct from the min-max or worst-case decision strategy [9]

Info-gap robustness does not require knowledge of a worst case. When even typical scenarios are poorly characterized, it is usually impractical to characterize worst cases, which is required by the min-max strategy. Info-gap theory does require specifying acceptable outcomes. Thus it is well suited to policy making, because preferences on outcomes are the driving force.

#### Info-gap robustness may proxy for the probability of satisfying the performance requirement [8,30,31]

A more robust option is often more likely to achieve the required outcome. By prioritizing the options using info-gap robustness, one maximizes the probability of satisfying the requirement, without knowing probability distributions. The proxy property is central to understanding survival in economic [8], biological [26] and other competitive environments [31].

### Info-gap implementation

Info-gap methodology requires three main elements: a *system model,* a *performance measure* and a *model of uncertainty*. The system model is a mathematical representation of a system and its influence on the variables of interest, for which management aspirations (performance criteria) are set. A performance measure assesses value or utility of outcomes. The model of uncertainty is a non-probabilistic representation of the degree to which the value of parameters, the form of a function, or the structure of a model may deviate from nominal estimates.

The system model in our example is summarized in two functions. *C*(*t*) is the variation over time of the total number of TB cases, untreated and treated, HIV-positive and HIV-negative, as a fraction of the initial population. *R*(*t*) is the total number of relapses, fast and slow, HIV-positive and HIV-negative, as a fraction of the initial population. (See eqs.(23) and (24) in Appendix “The Murray-Salomon model” section.)

The public health practitioner wishes to control the total number of TB cases: the fewer the better. However, trying to minimize this prevalence depends on model predictions that are highly uncertain. The performance requirement is to keep the total fraction of TB cases at a specified time, *t*_m_, below a critical value, *C*_m_, eq.(25) in Appendix “Performance requirements” section.

Grassly *et al*[32] note, in discussing epidemiology of HIV/AIDS, that “not all sources of error are amenable to statistical analysis” (p.i37), due to biased, inaccurate or unavailable data. The basic idea of info-gap model uncertainty is that we do not know how wrong our estimates are, we have no reliable knowledge of worst cases, and we do not know probability distributions for the estimates. The info-gap model uncertainty model is a non-probabilistic quantification of uncertainties.

A dominant uncertainty in TB dynamics with HIV prevalence is in model parameter values, though HIV causes significant uncertainties in model structure. Structural uncertainty refers to missing terms in the equations, missing equations, or unknown nonlinearities. Structural uncertainty is dealt with much less frequently than parameter uncertainty because of technical challenges. We focus on parameter uncertainty in this paper because of its importance and to facilitate the presentation of this first application of info-gap theory to public health.

We use info-gap theory [8] to model and manage uncertainties in the following parameters: slow and fast relapse rates for HIV positives and negatives, TB infection rates for HIV positives and negatives, and the HIV infection rate. Much literature suggests these parameters for their impact on the course of epidemics and the difficulty in measuring them [10,11,16,33-36]. Other uncertainties could also be investigated, depending on the purpose of the analysis. We use estimated values for each uncertain parameter, and estimated errors typically chosen as half of an interval estimate of the parameter. The info-gap model of uncertainty is specified in Appendix “Uncertainty” section.

We aim to achieve the performance requirement by judicious choice of control variables, defined in Appendix “Control variables” section. Eligible control variables are any coefficients of the dynamic model that can be influenced by public health or related medical intervention. We use the diagnosis rate, cure rate, relapse rate, and HIV infection rate. We define an intervention in terms of the values of these variables [15,34,37-40].

## Results: robustness and policy evaluation

We use the info-gap robustness function to evaluate alternative interventions aimed at controlling the relative TB prevalence, *C*(*t*), at a specified target time, *t*_m_, in the future. An intervention is specified by the values of the control variables. The evaluation leads to realistic assessment of outcomes and preferences among the interventions.

### Interpreting robustness curves: trade off and zeroing

All info-gap robustness curves have two properties, mentioned earlier: trade off between performance and robustness, and zeroing of the robustness curve. These properties are central in using robustness curves to evaluate public health policy.

**Figure 1:**
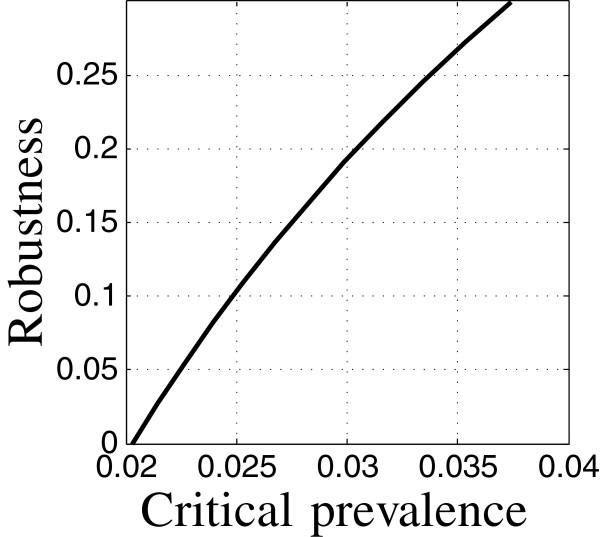
**Robustness of relative TB prevalence.** Run 8.

The coefficients of the epidemiological models are specified in Tables 1 and 2. Thoughout our examples, the initial conditions correspond to low TB and low HIV prevalence (the first data-column of Table 3) unless specified otherwise. The control variables specified in Appendix “Control variables” section are themselves model parameters. The robustness curve in Figure 1 is evaluated for the nominal values of the control variables specified in Tables 1 and 2. This set of control variables is the “baseline intervention”. The uncertain variables specified in Appendix “Uncertainty” section are also model parameters. Their nominal values and uncertainty estimates are specified in Table 4. These nominal values are the same as appear in Tables 1 and 2 for these variables. The total case load is evaluated at time *t*_m_=10 years after initiation unless indicated otherwise.

**Table 1:** Model parameters in the Murray-Salomon basic model

**Symbol**	**Definition**	**HIV neg**	**HIV pos**
Birth rate ^g^	births/year/person	0.03^*c*^	0^*c*,*h*^
*N*	population size	0.821^*i*^	0.179^*i*^
*T*	births per year = birth rate×*N*^*c*^		
*λ*^g^	infection rate	1.81×10^−3^^*m*^	2.96×10^−3^^*m*^
*K*^*k*^	# respiratory contacts with infected/person/year		
*L*^*k*^	probability that respir. contact with infectious source leads to infection		
*KL*		5–15^*a*^	
*Y*^*k*^	# infectious cases in population		
*μ*^g^	non-TB death rate	0.009^*c*^	0.05^*c*^
*p*	proportion of new infections entering slow breakdown	0.9 (0.85–0.95)^*a*^	0.4 (0.3–0.5^*a*^)
*β*_*F*_^*g*^	fast breakdown rate	2^*c*^	3
*β*_*S*_^g^	slow breakdown rate	0.001 (5–15×10^−4^^*a*^)	0.075 (0.05–0.10^*a*^)
*χ*^g^	rate of application of INH to infected individuals	0.75 (^*ℓ*^)	
*ν*	protection from superinfection conferred by primary infection	0.75 (0.5–1^*a*^)	
*w*	short-term INH effectiveness	0.7	
*h*	long-term INH effectiveness	0.7	
*d*^*i*,*j*^	proportion of pre-diagnosed cases in clinical category *i* entering diagnosis category *j*		
*d*^1,1^		0.45 (0.4–0.5)^*j*^	0.35 (0.3–0.4)^*j*^, ^*e*^
*d*^2,1^		0.55 (0.5–0.6)	0.65 (0.6–0.7) ^*e*^
*d*^3,1^			^*e*^
*d*^*i*,2^		*d*^*i*,2^=1−*d*^*i*,1^	^*f*^
*s*^*i*^	proportion of new cases in clinical category *i*		
*s*^1^, *s*^2^	proportion of new cases in clinical category *i*	0.45 (0.4–0.5^*a*^)	
*s*^3^	proportion of new cases in clinical category *i*	*s*^3^=1−*s*^1^−*s*^2^^*d*^	
*δ*^*j*^^g^	diagnosis rate for category *j*	0.6 ^*ℓ*^	0.6 ^*ℓ*^
*σ*^g^	smear conversion rate	0.03 ^c^	

**Model parameters** in the Murray-Salomon basic model, Table Two, p.42, in ref. [18], except for *K*, *L*, *Y* and *N* which are defined in footnote 2 on p.21 of [18]. Where a value is specified only for HIV-negative, the same value is used for HIV-positive.

^a^Footnote 8, p.24, [18].

^b^Footnote e, Table A5, p.63, [18].

^c^Table A5, p.63, [18].

^d^Footnote c, Table A5, p.63, [18].

^e^Footnote *†*, Table A5, p.63, and Figure A5, p.57 [18].

^f^Footnote b, Table A5, p.63,[18].

^g^Rate: per person per year. In Botswana the average is 477 cases per year per 100,000 people. 62% of them are HIV infected.

^h^Depends on HIV prevalence. In areas with HIV and without preventive treatment, 25% of babies born from HIV mothers are infected.

^i^In Botswana.

^j^[Dye C, Scheele S, Dolin P, Pathania V, Raviglione MC. Consensus statement. Global burden of tuberculosis: estimated incidence, prevalence, and mortality by country. WHO Global Surveillance and Monitoring Project. *JAMA.* 1999 Aug 18;282(7):677–86.].

^k^Not needed since *λ*is a primary datum.

^ℓ^Decision variable.

^m^Can also be treated as a decision variable.

**Table 2:** Model parameters in the Murray-Salomon basic model

**Symbol**	**Definition**	**HIV neg**	**HIV pos**
*ϵ*_*U*_^*f*^	spontaneous cure rate for untreated cases	0.2 (0.14–0.25)^*g*^	
$\mu_{U}^{i}$^*f*^	TB death rate for untreated cases in clinical category *i*		
$\mu_{U}^{1}$		0.12 (0.075–0.20^*a*^)	0.45 (0.3–0.6^*a*^)
$\mu_{U}^{2}$, $\mu_{U}^{3}$		$0.7\mu_{U}^{1}$^*b*^	$0.7\mu_{U}^{1}$^*b*^
*g*^*i*,*k*^	proportion of treated cases in clinical category *i* and treatment category *k*		
*g*^1,1^		0.5 ^*d*^	
*g*^2,1^		0.28 ^*d*^	
*g*^3,1^		^*d*^	
*g*^*i*,2^		*g*^*i*,2^=1−*g*^*i*,1^^*e*^	
$\epsilon_{T}^{k}$^*f*^	cure rate for treated case in treatment category *k*		
$\epsilon_{T}^{1}$		0.8	
$\epsilon_{T}^{2}$		0.5^*c*^	
$\mu_{T}^{i,k}$^*f*^	TB death rate for treated cases in clinical category *i* and treatment category *k*		
$\mu_{T}^{1,1}$		0.075^*c*^	0.16^*c*^
$\mu_{T}^{1,2}$		0.12^*c*^	0.24^*c*^
$\mu_{T}^{2,k}$, $\mu_{T}^{3,k}$		$0.7\mu_{T}^{1,k}$^*b*^	$0.7\mu_{T}^{1,k}$^*b*^
*r*_*U*_	proportion of spontaneously recovered cases entering the slow relapse category	0.009^*c*^	
$r_{T}^{k}$	proportion of recovered cases from treatment category *k* entering the slow relapse category		
$r_{T}^{1}$		0.0096^*c*^	
$r_{T}^{2}$		0.0094^*c*^	
*ρ*_*F*_^*f*^	fast relapse rate	2^*c*^	3
*ρ*_*S*_^*f*^	slow relapse rate	0.001 (5–15×10^−4^^*a*^)	
*γ*^*f*^	rate of HIV infection	0.075 (0.011–0.95)^*h*^	

**Model parameters** in the Murray-Salomon basic model, Table Two, p.42, in ref. [18], except for *K, L, Y* and *N* which are defined in footnote 2 on p.21 of [18]. Where a value is specified only for HIV-negative, the same value is used for HIV-positive.

^a^Footnote 8, p.24, [18].

^b^Footnote e, Table A5, p.63, [18].

^c^Table A5, p.63, [18].

^d^Footnote *†*, Table A5, p.63, and Figure A4, p.56 [18].

^e^Footnote d, Table A5, p.63, [18].

^f^Rate: per person per year.

^g^[33,34]. Other estimate: 0.05–0.15^*a*^.

^h^[1].

**Table 3:** Initial conditions

	**Symbol**	**Low TB Prevalence**			**Medium TB Prev.**			**High TB Prev.**		
		**Low**	**Med**	**High**	**Low**	**Med**	**High**	**Low**	**Med**	**High**
		**HIV**	**HIV**	**HIV**	**HIV**	**HIV**	**HIV**	**HIV**	**HIV**	**HIV**
1	*U*	0.9	0.8	0.6	0.8	0.650	0.500	0.6	0.5	0.4
2	*I*_*F*_	0.0075	0.015	0.03	0.011	0.018	0.028	0.03	0.0375	0.045
3	*I*_*S*_	0.03	0.06	0.12	0.05	0.088	0.125	0.12	0.15	0.18
4	*S*_*F*_	0.003	0.006	0.012	0.01	0.018	0.025	0.012	0.015	0.018
5	*H*_*S*_	0.009	0.018	0.036	0.02	0.035	0.050	0.036	0.045	0.054
	$C_{U}^{i,j}$									
6	(*i*,*j*)=(1,1)	0.005	0.01	0.02	0.008	0.014	0.020	0.02	0.025	0.03
7	(*i*,*j*)=(2,1)	0.002	0.004	0.008	0.003	0.005	0.008	0.008	0.01	0.012
8	(*i*,*j*)=(3,1)	0.002	0.004	0.008	0.003	0.005	0.008	0.008	0.01	0.012
9	(*i*,*j*)=(1,2)	0.002	0.004	0.008	0.003	0.005	0.008	0.008	0.01	0.012
10	(*i*,*j*)=(2,2)	0.001	0.002	0.004	0.002	0.004	0.005	0.004	0.005	0.006
11	(*i*,*j*)=(3,2)	0.001	0.002	0.004	0.002	0.004	0.005	0.004	0.005	0.006
	$C_{T}^{i,k}$									
12	(*i*,*k*)=(1,1)	0.01	0.02	0.04	0.02	0.035	0.050	0.04	0.05	0.06
13	(*i*,*k*)=(2,1)	0.005	0.01	0.02	0.01	0.018	0.025	0.02	0.025	0.03
14	(*i*,*k*)=(3,1)	0.005	0.01	0.02	0.01	0.018	0.025	0.02	0.025	0.03
15	(*i*,*k*)=(1,2)	0.005	0.01	0.02	0.02	0.035	0.050	0.02	0.025	0.03
16	(*i*,*k*)=(2,2)	0.002	0.004	0.008	0.005	0.009	0.013	0.008	0.01	0.012
17	(*i*,*k*)=(3,2)	0.0025	0.005	0.01	0.003	0.005	0.008	0.01	0.0125	0.015
18	*R*_*F*_	0.002	0.004	0.008	0.005	0.009	0.013	0.008	0.01	0.012
19	*R*_*S*_	0.006	0.012	0.024	0.015	0.025	0.038	0.024	0.03	0.036
		1.000	1.000	1.000	1.000	1.000	1.000	1.000	1.000	1.000

**Table 4:** Nominal values and error weights of uncertain variables

**Symbol**	**Nominal value,**${\tilde{u}}_{i}$	**Error weight,*s***_***i***_
*λ*	1.81×10^−3^	0.0009
$\overset{Â¯}{\lambda }$	2.96×10^−3^	0.0018
*γ*	0.075	0.2
*ρ*_*S*_	0.001	0.0005
${\overset{Â¯}{\rho }}_{S}$	0.001	0.001
*ρ*_*F*_	2	1
${\overset{Â¯}{\rho }}_{F}$	3	1.5

**Figure 2:**
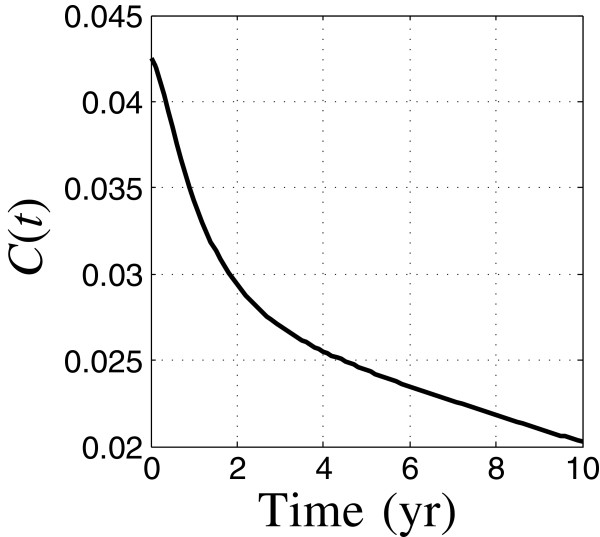
**Relative TB prevalence vs. time.** Run 8.

**Figure 3:**
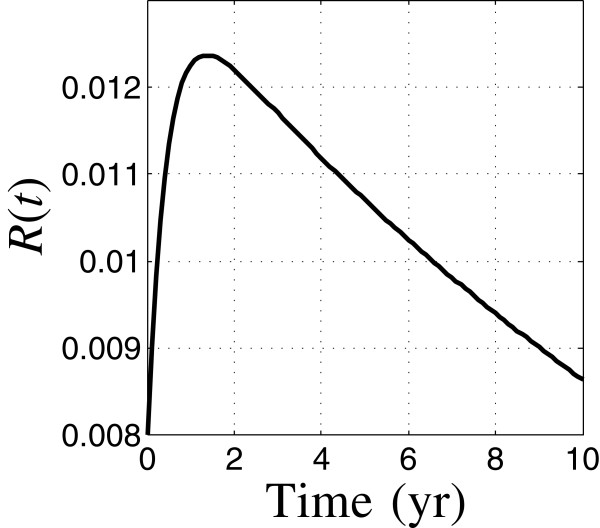
**Relative relapses vs. time.** Run 8.

Figures 2 and 3 show the temporal evolution of the relative prevalence of TB cases, *C*(*t*), and relative relapses, *R*(*t*), based on the nominal estimates of the model parameters, with moderately low initial TB and HIV prevalence. *C*(*t*) and *R*(*t*) are fractions of the initial total population size. Figure 2 shows that the total number of TB cases starts at about 4.2% of the initial population and decays to about 3% in the first 1.5 years, thereafter decaying more slowly, reaching 2.1% of the initial population size after 10 years. The relapse population starts very small, rises rapidly in the first year and thereafter decays gradually. The reduction in the rate of decrease of the TB cases after 1.5 years, Figure 2, results from the influx of relapses which have built up since initiation of the intervention.

#### Trade off

Key to understanding the trade off expressed by the robustness curve is the concept of satisficing. In contrast to optimizing, satisficing asks for an outcome that meets minimal needs but may not be the best imaginable. The satisficing strategy is not merely “accepting second best.” Satisficing is aspirational, setting a goal just like optimization, but also requiring robustness to uncertainty. The satisficing strategy induces a trade off between the aspiration for good outcome and the robustness against uncertainty in attaining that outcome.

The robustness curve in Figure 1 is based on satisficing the relative TB prevalence: requiring that the prevalence not exceed the critical value, *C*_m_. Figure 1 shows the robustness vs. the critical prevalence. The positive slope of the robustness curve in Figure 1 expresses the trade off between robustness and performance: large robustness entails large prevalence at the specified target time (10 years). Equivalently, requiring low relative prevalence entails low robustness to uncertainty in the epidemiological model. The robustness curve quantifies the intuition that more demanding outcomes (small prevalence) are more vulnerable to model uncertainty (small robustness).

We can interpret the numerical values along the robustness curve as follows. The prevalence, *C*(*t*), and its critical value, *C*_m_, are normalized to the initial population size. For instance, *C*_m_=0.025 means that the prevalence at time *t*_m_ must not exceed 2.5% of the initial population size. The robustness corresponding to this value of *C*_m_, is 0.1 as seen in Figure 1. This means that the performance requirement is guaranteed if the uncertain model parameters vary from their nominal values by no more than 10% of their error estimates. (The model parameters are constrained to be positive since they are first-order rate constants.)

The public health practitioner may feel that robustness to 10% uncertainty in the model parameters is rather small, given the substantial uncertainty in the epidemiological dynamics of TB with HIV prevalence. If we want robustness to, say, 25% uncertainty in the model parameters we must accept a larger final case load, namely, *C*_m_=0.033 as seen in Figure 1. Greater robustness is obtained only by accepting poorer outcome; this is an irrevocable trade off that is quantified by the robustness curve.

#### Zeroing

We note that the robustness curve in Figure 1 reaches the horizontal axis at the value *C*_m_=0.021. This means that requiring the prevalence not to exceed 2.1% of the initial population has no robustness against model uncertainty. The value of *C*_m_ at which the robustness becomes zero is precisely the nominal prediction of the prevalence at time *t*_m_ as seen by the right end-point in Figure 2. That is, the value of *C*(*t*_m_), evaluated with the best estimates of the model parameters, equals 0.021. The horizontal intercept in Figure 1 is an example of the property of zeroing that holds for all info-gap robustness curves: The outcome predicted by the model, when adopted as the performance requirement, has no robustness against uncertainty in the model.

It is not surprising that the predicted outcome is extremely vulnerable to error in the model upon which the prediction is based. However, the zero-robustness of predicted outcomes has an important implication for policy selection.

The robustness curve in Figure 1 is for a particular choice of values of the control variables: the baseline intervention. The zeroing property—no robustness of the predicted outcome of these control values—implies that we should not assess these control values in terms of their predicted outcome. The predicted prevalence of 0.021 at time *t*_m_=10 years does not reliably reflect the performance of these control variables. Due to the trade off property, only larger prevalence can reliably be expected to result from this choice of the control variables. Predicted outcomes are not reliable for prioritizing the interventions.

### Equivalent interventions

**Figure 4:**
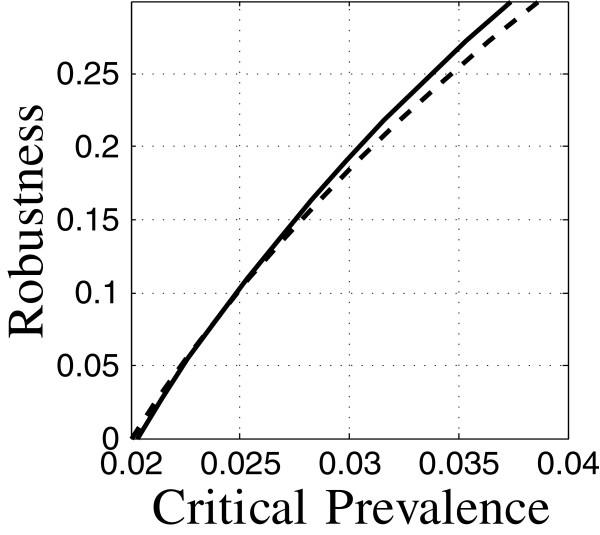
**Equivalent robustness for two interventions.** Run 8: —, run 15: – –.

**Figure 5:**
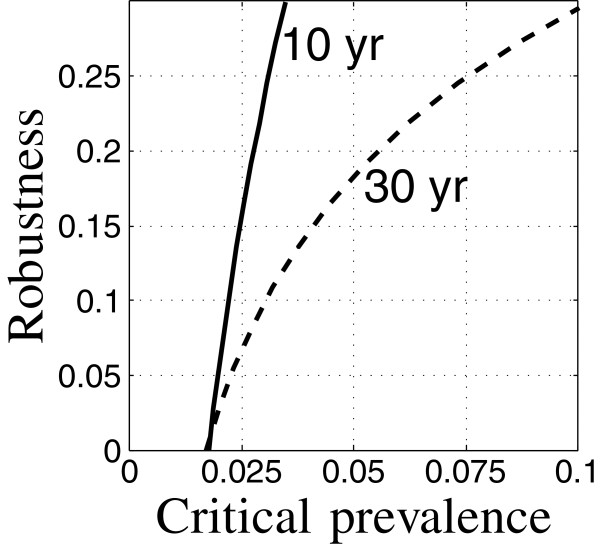
**Robustness curves at 10, 20 and 30 years.** Run 8: —, run 27: – –, run 28: ·–.

Different combinations of interventions can yield essentially equivalent results, as in Figure 4. The baseline intervention (solid), is characterized by low diagnosis rate and high relapse rate. The other intervention (dash) has higher diagnosis rate and lower relapse rate as specified in Table 5. (Interventions are specified by the values of control variables presented in Table 5). The robustness curves for these two control strategies, at 10 years, are nearly the same, suggesting that the public health practitioner may choose freely between them, perhaps employing additional criteria such as cost or ease of implementation. Equivalence may be lost if parameters are changed. For instance, we will see later (Figure 5) that these interventions evaluated at 10, 20 or 30 years have very different robustness curves.

**Table 5:** Control variables for robustness curves

**Run**	**Init**	***t***_**m**_	$\delta^{j}={\overset{Â¯}{\delta }}^{j}$	$\epsilon_{T}^{k}={\overset{Â¯}{\epsilon }}_{T}^{k}$	***ρ***_***F***_	${\overset{Â¯}{\rho }}_{F}$	***λ***	$\overset{Â¯}{\lambda }$	***γ***	***β***_***F***_	${\overset{Â¯}{\beta }}_{F}$	
	**Prev**^**a**^	**(yr)**										
8	1	10	(0.6, 0.6)	(0.8, 0.5)	2	3	0.00181	0.00296	0.075	2	3	
9	1	10	(0.65, 0.65)	(0.8, 0.5)	2	3	0.00181	0.00296	0.075	2	3	
10	1	10	(0.65, 0.65)	(0.88, 0.55)	2	3	0.00181	0.00296	0.075	2	3	
11	1	10	(0.65, 0.65)	(0.88, 0.55)	1.5	2.25	0.00181	0.00296	0.075	2	3	
12	1	10	(0.65, 0.65)	(0.88, 0.55)	1	1.5	0.00181	0.00296	0.075	2	3	
15	1	10	(0.85, 0.85)	(0.8, 0.5)	1.2	2	0.00181	0.00296	0.075	2	3	
19	5	10	(0.6, 0.6)	(0.8, 0.5)	2	3	0.00181	0.00296	0.075	2	3	
21	5	10	(0.65, 0.65)	(0.8, 0.5)	2	3	0.00181	0.00296	0.075	2	3	
22	5	10	(0.65, 0.65)	(0.88, 0.55)	2	3	0.00181	0.00296	0.075	2	3	
23	5	10	(0.65, 0.65)	(0.88, 0.55)	1	1.5	0.00181	0.00296	0.075	2	3	
20	9	10	(0.6, 0.6)	(0.8, 0.5)	2	3	0.00181	0.00296	0.075	2	3	
24	9	10	(0.65, 0.65)	(0.8, 0.5)	2	3	0.00181	0.00296	0.075	2	3	
25	9	10	(0.65, 0.65)	(0.88, 0.55)	2	3	0.00181	0.00296	0.075	2	3	
26	9	10	(0.65, 0.65)	(0.88, 0.55)	1	1.5	0.00181	0.00296	0.075	2	3	
27	1	20	(0.6, 0.6)	(0.8, 0.5)	2	3	0.00181	0.00296	0.075	2	3	
28	1	30	(0.6, 0.6)	(0.8, 0.5)	2	3	0.00181	0.00296	0.075	2	3	
29	1	10	(0.6, 0.6)	(0.8, 0.5)	2	3	0.00181	0.00296	0.0375	2	3	
30	1	10	(0.6, 0.6)	(0.8, 0.5)	2	3	0.00181	0.00296	0.05	2	3	
31	1	10	(0.6, 0.6)	(0.8, 0.5)	2	3	0.00181	0.00296	0.06	2	3	
32	1	10	(0.6, 0.6)	(0.8, 0.5)	2	3	0.0009	0.00148	0.075	2	3	
33	1	10	(0.6, 0.6)	(0.8, 0.5)	2	3	0.0003	0.0005	0.075	2	3	
38	1	30	(0.85, 0.85)	(0.8, 0.5)	2	3	0.00181	0.00296	0.075	2	3	
39	1	10	(0.6, 0.6)	(0.8, 0.5)	2	3	0.00181	0.00296	0.075	1	1.5	
41	1	10	(0.65, 0.65)	(0.8, 0.5)	2	3	0.00181	0.00296	0.075	1	1.5	

^a^Data-column in Table 3.

**Figure 6:**
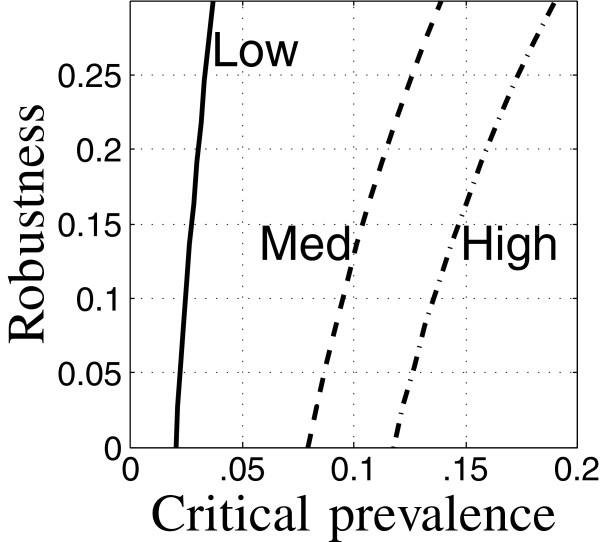
**Nominal equivalence of two interventions.** Run12: —, run 38: – –.

Figure 6 shows a different aspect of the equivalence of interventions. The figure shows robustness curves for two strategies specified in Table 5. Both strategies aim to control the relative prevalence of TB, but one (solid) is geared for a 10-year target time, while the other (dash) considers a 30-year target. The estimated outcomes—prevalence—are very nearly the same for these two strategies, each at its respective target time, as shown by their shared horizontal intercept at *C*_m_=0.018. These predictions result from estimated model parameters, so one might be inclined to conclude that TB prevalence of 0.018 can be achieved at either 10 or 30 years by using the corresponding intervention.

However, the epidemiological model is highly uncertain, and the robustness curves in Figure 6 of these two strategies are quite different. Not surprisingly, the 30-year target is much less robust to uncertainty. It would be erroneous to treat these two strategies as outcome-equivalent since their performances at positive robustness are quite different. Nominal equivalence (equivalence of the predicted outcome) does not imply robustness equivalence.

### Impact of initial TB and HIV prevalence

We now consider higher initial prevalences. The overall shape of the dynamic response is very similar in each case, except that the prevalence increases significantly as the initial prevalence increases. As in Figures 2 and 3, in each scenario the initial TB prevalence decreases rapidly during the first 2 years, and thereafter decreases more slowly as the new relapse population—which peaks around the end of the first year—flows back into active cases.

**Figure 7:**
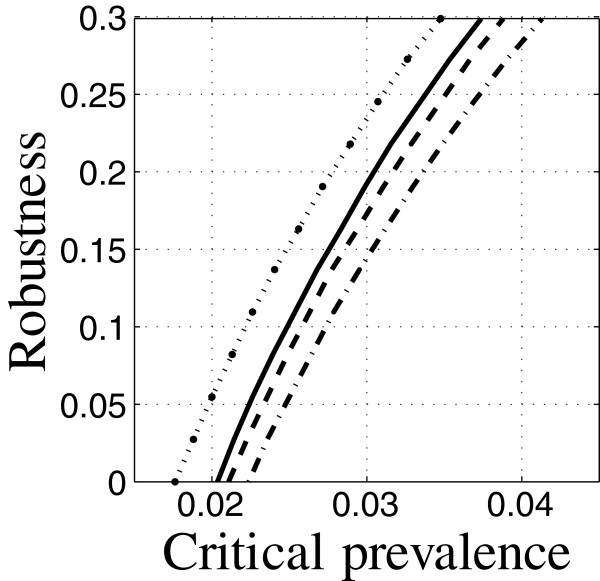
**Robustness curves for low, medium and high initial TB and HIV prevalence.** Run 8: —, run 19: – – run 20: ·–.

Figure 7 shows robustness curves for a target time 10 years after initiation, for low (solid), medium (dash) and high (dot-dash) initial prevalence of TB and HIV. The low-prevalence curve (solid) is the same as Figure 1. The robustness curves shift dramatically to the right as the baseline prevalence of TB and HIV increases, indicating poorer estimated outcome and lower robustness to uncertainty.

### Intervention aggressiveness

**Figure 8:**
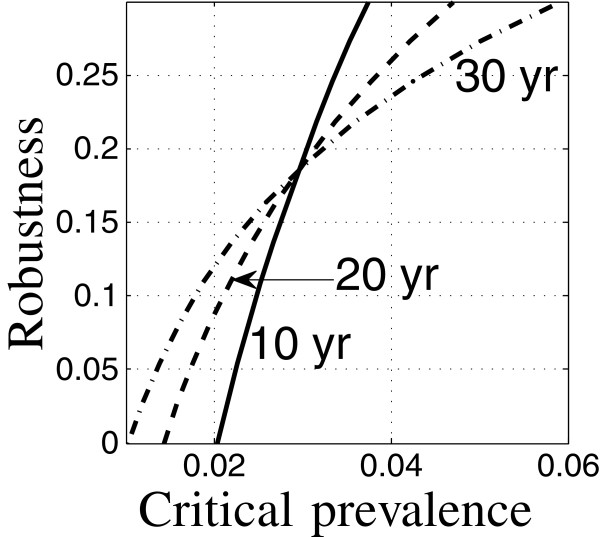
**Robustness with varying aggressiveness.** Run 8: —, run 9: – –, run 10:·–, run 12: ⋯.

Figure 8 shows robustness curves for low initial TB and HIV prevalence with interventions specified in Table 5. The solid curve is the baseline intervention, against which the other curves entail more aggressive intervention in either or both the active cases and the relapse population.

The progression from solid to dash to dot-dash in Figure 8 represents increasingly aggressive intervention in the active TB case population. We see that increasing aggressiveness, in this specific parameter configuration, results in increasing prevalence and decreasing robustness to model error at the target time. The explanation is that aggressive treatment of active cases enlarges the relapse population which flows back into the active case population.

The top curve in Figure 8 modifies the most aggressive case (dot-dash) by also including more aggressive intervention in the TB relapse population. This reduction in relapse reduces the predicted prevalence after 10 years, and increases the robustness to uncertainty.

### Different target times

Most of the results discussed so far evaluated the robustness for a target time 10 years after initiation. We now consider the implications of different target times.

Figure 5 shows robustness curves at target times, *t*_m_, of 10, 20 and 30 years (solid, dash, dot-dash respectively). The initial prevalences of TB and HIV are low. The interventions are all at the baseline.

The predicted prevalence decreases as the target time increases, as shown by the horizontal intercepts in Figure 5. The baseline intervention is predicted to reduce the prevalence, (in units of initial population size), as the time horizon increases. However, the zeroing property means that these predictions have no robustness to uncertainty in the model used for prediction. Only higher prevalence has positive robustness.

From Figure 5 we see that, for critical TB prevalence *C*_m_ less than 3%, the 30-year TB prevalence is more robust than the 20-year prevalence which is more robust than the 10 year prevalence. For instance, at critical TB prevalence of *C*_m_=0.02, the robustnesses for 10, 20 and 30 year horizons are 0, 0.08 and 0.12, respectively. This intervention has no robustness to uncertainty when requiring a 2% prevalence after 10 years; in fact, the estimated prevalence at 10 years is greater than 2%. The prevalence at 20 years will be no worse than 2% provided that the model coefficients err by no more than 8%, and at 30 years the robustness to error is 12%.

The practitioner may feel that even 12% robustness against model-coefficient error is rather small, given the severe uncertainty of TB epidemiology in the context of epidemic HIV. This means that, even at a 30-year horizon, this intervention cannot reliably achieve a relative prevalence as low as 2%.

Suppose we are willing to aim at a final TB prevalence of 3.7%. We see from Figure 5 that now the 10-year horizon is more robust than 20 years which is more robust than 30 years. The robustnesses are now 30%, 24% and 22% for 10, 20 and 30 years. The robustness curves have intersected one another and the robustness rankings are reversed. As the target time decreases, the predicted outcome becomes worse (horizontal intercept moves right) but the cost of robustness improves. This causes the robustness curves to cross one another. More intuitively, we can say that prediction of TB prevalence is more reliable for short time horizon than for long times. But since a long time is required to overcome the relapse effect, we observe the intersection of the robustness curves and the consequent reversal of their robust dominance.

Results like Figure 5 have important policy implications for TB control over long time periods. The policy maker may be tempted to choose one option that is predicted to yield better short term results. However, that choice might be wrong when one opts to satisfice the outcome with robustness to uncertainty. Predictions of mathematical models (horizontal intercepts) are not sufficiently reliable for comparing and prioritizing interventions; the cost of robustness (slope) must also be considered. In the example in Figure 5 one might conclude that prevalence less than 3% is not achievable at any target time, that 3.7% is feasible at 10-years but not beyond, and that other interventions are needed for longer-term outcomes.

### Impact of HIV mortality

**Figure 9:**
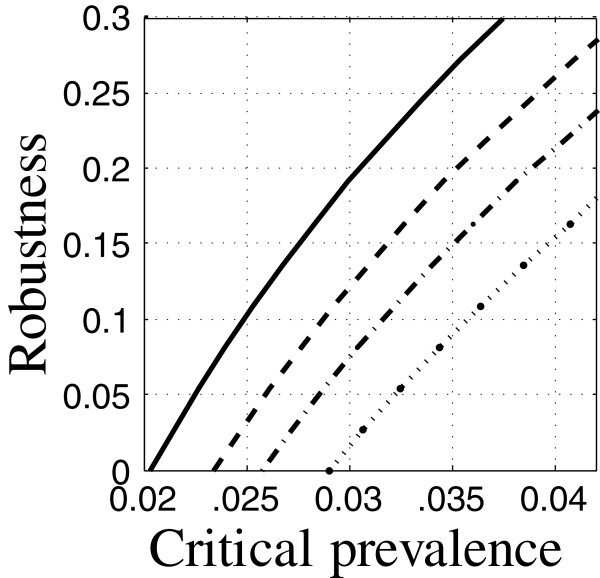
**Robustness for various HIV infection rates.** Run 8: —, run 31: – –, run 30: ·–, run 29: ⋯.

Figure 9 shows 10-year robustness curves for various HIV infection rates, with low initial TB and HIV prevalence, as specified in Table 5. The HIV infection rate decreases in the progression from solid, dash, dot-dash to dot-dot. As the HIV infection rate decreases, the estimated 10-year TB prevalence increases and the robustness decreases. The explanation lies in the high mortality rate of the HIV population. As the HIV infection rate decreases, the size of the relapse population decays more slowly, allowing greater flow back into the active TB case population. Interventions that decrease HIV infection rates or restore immunity to HIV patients, will counter-intuitively tend to increase TB prevalence unless compensating measures are taken. Significantly, the cost of robustness (slope of the robustness curve) does not change as a result of decreased HIV infection rate. Reducing HIV infection rate shifts the robustness curve to the right, with almost no change in slope.

## Conclusion

We demonstrated a generic info-gap framework for managing model uncertainty in public health decision making. By applying it to a mathematical model of TB/HIV epidemics, we illustrated specific recommendations for interventions in the control of TB with HIV in various settings.

The complicated multi-dimensional epidemiological dynamics are dominated by the flow back and forth between the actively and latently infected TB populations and the different rates of progression of different subpopulations between these compartments. Counter-intuitively, the total TB case load even decades after initiation can increase as a result of increased diagnosis and cure rates, and it can increase as the control of HIV becomes more aggressive. These findings highlight the critical importance of modeling in the assessment and planning of public health intervention. Model predictions are often used to choose interventions. However, model predictions must be interpreted in light of model uncertainties. Predicted outcomes have zero robustness to model error. Only worse-than-predicted outcomes (higher relative prevalence) have positive robustness against model error. This means that predicted outcomes are not reliable for prioritizing the interventions. The trade off between robustness and outcome is quantified by the info-gap model analysis and is a critical component of the decision-making process.

We explore the performance of interventions that alter the rate constants of diagnosis, cure, relapse and HIV infection. Some interventions have quite similar predicted outcomes and robustness curves. This enables the policy maker to choose between these interventions based on additional criteria, such as ease or cost of implementation. It is not true, however, that interventions with the same estimated outcomes necessarily have the same robustness against model error.

We demonstrate the policy implications of initial TB and HIV prevalence, of HIV mortality, of degree of treatment aggressiveness, and of the target time at which outcomes are evaluated. Public health policies are evaluated in terms of confidence—expressed as robustness to modeling error—in achieving specified TB prevalence at the target time. Predicted outcomes have zero robustness and thus are not reliable for evaluating and comparing interventions. Instead, interventions must be prioritized in terms of their capacity for achieving specified outcomes, with robustness to uncertainty. Failure to quantify the uncertainty inherent in public health interventions leads to disappointment from unrealized expectations, and failed policy. Where a public health model underlies guidelines, info-gap decision theory provides valuable insight into the confidence of achieving agreed-upon goals.

## Appendices

### The Murray-Salomon model

The Murray-Salomon (M-S) model [17,18] is a set of coupled differential equations that describe the time evolution of TB. A modification deals with TB infecteds in a population containing HIV smear-positive individuals. In section “The basic Murray-Salomon model: No HIV” we define the basic non-HIV model. In section “The HIV-Extended model” we present the M-S extension to include an HIV sub-population. The state variables are defined in Table 6 and the parameters are defined in Table 1.

**Table 6:** **State variables—sizes of sub-populations—in the Murray-Salomon basic model, Table One, p.41, in ref. [**[18]**]**

**Index**	**Symbol**	**Definition**	**Initial value**^**a**^		
			**HIV Neg**	**HIV pos**	**Ref.**
1	*U*	Uninfected	0.9^b^	0.2^c^	[10]
2	*I*_*F*_	Infected subject to fast breakdown	0.05–0.1	0.1	[33,34]
3	*I*_*S*_	Infected subject to slow breakdown	0.1	0.1	[33,34]]
4	*S*_*F*_	Superinfected subject to fast breakdown	0		
5	*H*_*S*_	INH recipient subject to slow breakdown	0.01	0.01–0.03	
6–11	$C_{U}^{i,j}$	Untreated cases, of 6 types:	0		
		*i*=1: smear-positive pulmonary			
		*i*=2: smear-negative pulmonary			
		*i*=3: extra-pulmonary			
		*j*=1: fast diagnosis category			
		*j*=2: slow diagnosis category			
		*i*^⋆^=2 if *i*=1 (eqs.(6) and (7))			
		*i*^⋆^=1 if *i*=2 (eqs.(6) and (7))			
		*i*^⋆^=0 if *i*=3 (eqs.(6) and (7))			
12–17	$C_{T}^{i,k}$	Treated cases, of 6 types:	0		
		*i* as above			
		*k*=1: good treatment category			
		*k*=2: bad treatment category			
18	*R*_*F*_	Recovered cases subject to fast relapse	0.4 (0.28–0.52)		[33,34]
19	*R*_*S*_	Recovered cases subject to slow relapse	0.050 (0.035–0.065)		[33,34]

The definition of superscript *i*^⋆^is in [18] on p.20.

^a^As fraction of total population at start of simulation.

^b^Low prevalence.

^c^High prevalence.

#### The basic Murray-Salomon model: No HIV

The basic M-S model is the following 19 differential equations (eqs.(6) and (7) occur in 6 different forms each) appearing on pp.19–20 of Murray and Salomon [18]:

(1) $$\frac{\text{d}U}{\text{d}t}=T-\mathrm{\lambda U}-\mathrm{\mu U}$$

(2) $$\frac{\text{d}I_{F}}{\text{d}t}=(1-p)\mathrm{\lambda U}-\beta_{F}I_{F}-\mathrm{w\chi}I_{F}-\mu I_{F}$$

(3) $$\frac{\text{d}I_{S}}{\text{d}t}=\mathrm{p\lambda U}-\beta_{S}I_{S}-\chi I_{S}-(1-p)\lambda (1-\nu )I_{S}-\mu I_{S}$$

(4) $$\frac{\text{d}S_{F}}{\text{d}t}=(1-p)\lambda (1-\nu )(I_{S}+H_{S}+R_{S})-\beta_{F}S_{F}-\mathrm{w\chi}S_{F}-\mu S_{F}$$

(5) $$\begin{array}{l} \frac{\text{d}H_{S}}{\text{d}t}=\;\chi (wI_{F}+wS_{F}+I_{S})-(1-p)\lambda (1-\nu )H_{S}\\ \;-(1-h)\beta_{S}H_{S}-\mu H_{S} \end{array}$$

(6) $$\begin{array}{lll} \;\frac{\text{d}C_{U}^{i,j}}{\text{d}t}= & \left\{\beta_{F}(I_{F}+S_{F})+\beta_{S}[I_{S}+(1-h)H_{S}]\right)\; & \;\\ & \left(+\rho_{S}R_{S}+\rho_{F}R_{F}\right\}d^{i,j}s^{i}\; & \;\\ & -\delta^{j}C_{U}^{i,j}\pm \sigma C_{U}^{i^{⋆},j}-\epsilon_{U}C_{U}^{i,j}-(\mu +\mu_{U}^{i})C_{U}^{i,j},\;\\ & \;\;\;\;\;\;\text{for}\;i=1,2,3\;\text{and}\;j=1,2\; & \; \end{array}$$

(7) $$\begin{array}{lll} \;\frac{\text{d}C_{T}^{i,k}}{\text{d}t} & =g^{i,k}\underset{j=1,2}{\sum }\delta^{j}C_{U}^{i,j}\pm \sigma C_{T}^{i^{⋆},k}-\epsilon_{T}^{k}C_{T}^{i,k}-(\mu +\mu_{T}^{i,k})C_{T}^{i,k},\;\\ & \;\;\;\;\;\;\;\;\text{for}\;i=1,2,3\;\text{and}\;k=1,2\; & \; \end{array}$$

(8) $$\begin{array}{l} \;\frac{\text{d}R_{F}}{\text{d}t}=\;(1-r_{U})\epsilon_{U}\underset{\overset{i=1,2,3}{j=1,2}}{\sum }C_{U}^{i,j}+\underset{k=1,2}{\sum }\left[\left(1-r_{T}^{k}\right)\epsilon_{T}^{k}\underset{i=1,2,3}{\sum }C_{T}^{i,k}\right]\\ \;-\rho_{F}R_{F}-\mu R_{F}\; \end{array}$$

(9) $$\begin{array}{lll} \;\frac{\text{d}R_{S}}{\text{d}t}=\; & r_{U}\epsilon_{U}\underset{\overset{i=1,2,3}{j=1,2}}{\sum }C_{U}^{i,j}+\underset{k=1,2}{\sum }\left[r_{T}^{k}\epsilon_{T}^{k}\underset{i=1,2,3}{\sum }C_{T}^{i,k}\right]\; & \;\\ & -\rho_{S}R_{S}-(1-p)\lambda (1-\nu )R_{S}-\mu R_{S}\; \end{array}$$

The term ‘±*σ*’ appears in eqs.(6) and (7). M-S write:^a^

 It should be noted in the equations for $C_{U}^{i,j}$ and $C_{T}^{i,j}$ that the smear rate *σ*is multiplied by the number of individuals in the respective category *i*^⋆^, where *i*^⋆^=2 (smear-negative) for *i*=1 (smear-positive) and *vice versa,* and *i*^⋆^=*∅*for *i*=3 (extra-pulmonary). The term including *σ*is added for *i*=1, subtracted for *i*=2, and equal to 0 for *i*=3. The result of this formulation is that smear-negative patients convert to smear-positive at a rate of *σ*.

However, the ‘vice versa’ is a mistake. The correct equations for $C_{U}^{i,j}$ (with analogs for $C_{T}^{i,j}$) are:

(10) $$\frac{\text{d}C_{U}^{1,j}}{\text{d}t}=\cdots +\sigma C_{U}^{2,j}+\cdots$$

(11) $$\frac{\text{d}C_{U}^{2,j}}{\text{d}t}=\cdots -\sigma C_{U}^{2,j}+\cdots$$

(12) $$\frac{\text{d}C_{U}^{3,j}}{\text{d}t}=\cdots +0C_{U}^{i^{⋆},j}+\cdots$$

Eq.(10) states that smear-negative individuals join the smear-positive population at rate *σ*. Eq.(11) states that smear-negative individuals leave the smear negative population at rate *σ*. That way all individuals are accounted for.

The instantaneous rate of infection, *λ*in eq.(1), is defined by Murray and Salomon [18], p.21, as:

(13) $$\lambda =\mathrm{KL}\frac{Y}{N}$$

#### The HIV-Extended model

*Introduction*We will now formulate the extended dynamic model to include a differentiation between HIV-positive and HIV-negative populations. M-S do this also, and state [18], p.4 that they use “two sub-models—one for the HIV sero-negative population, and one for the HIV sero-positive population. Each sub-model follows the structure” which is presented here as eqs.(1)–(9). They write that 

 Individuals move from each category in the HIV-negative sub-model to the corresponding category in the HIV-positive sub-model at the HIV infection rate, which varies over time. Because the effects of HIV on immune function are not marked with respect to tuberculosis until the CD4 count has dropped below 500, we actually move individuals from the HIV-negative to the HIV-positive sub-model after they have been infected with HIV for 3 years. The two sub-models are also linked through the annual risk of infection, as HIV-negative tuberculosis cases can infect HIV-positive individuals, and vice versa [18], pp.4–5

Our model does not delay transfer from the HIV-negative sub-model.

*Sub-models* Each of the two sub-populations—HIV-negative and HIV-positive—is divided into the 19 groups represented by the state variables in Table 6. Each state variable has a differential equation in eqs.(1)–(9).

Let us denote the HIV-negative state variables as before, and the HIV-positive state variables with the same letters but with an over-bar. For compactness we represent these two sets of variables with two vectors:

(14) $$\begin{array}{lll} x= & \left(U,I_{F},I_{S},S_{F},H_{S},C_{U}^{1,1},C_{U}^{2,1},C_{U}^{3,1},C_{U}^{1,2},C_{U}^{2,2},C_{U}^{3,2},\right)\; & \;\\ & \;\left(C_{T}^{1,1},C_{T}^{2,1},C_{T}^{3,1},C_{T}^{1,2},C_{T}^{2,2},C_{T}^{3,2},R_{F},R_{S}\right)\; \end{array}$$

(15) $$\begin{array}{lll} \overset{Â¯}{x}= & \;\left(\overset{Â¯}{U},{\overset{Â¯}{I}}_{F},{\overset{Â¯}{I}}_{S},{\overset{Â¯}{S}}_{F},{\overset{Â¯}{H}}_{S},{\overset{Â¯}{C}}_{U}^{1,1},{\overset{Â¯}{C}}_{U}^{2,1},{\overset{Â¯}{C}}_{U}^{3,1},{\overset{Â¯}{C}}_{U}^{1,2},{\overset{Â¯}{C}}_{U}^{2,2},{\overset{Â¯}{C}}_{U}^{3,2},\right)\; & \;\\ & \left(\;{\overset{Â¯}{C}}_{T}^{1,1},{\overset{Â¯}{C}}_{T}^{2,1},{\overset{Â¯}{C}}_{T}^{3,1},{\overset{Â¯}{C}}_{T}^{1,2},{\overset{Â¯}{C}}_{T}^{2,2},{\overset{Â¯}{C}}_{T}^{3,2},{\overset{Â¯}{R}}_{F},{\overset{Â¯}{R}}_{S}\right)\; \end{array}$$

The model parameters listed in Tables 1 and 2 take different values for HIV-negative and HIV-positive populations (as specified in the tables). Let us denote the model parameters as before for the HIV-negative population, and use the same symbols with an over-bar for the HIV-positive population.

Eqs.(1)–(9) are 1st-order linear inhomogeneous differential equations. Only eq.(1) has an inhomogeneous term: *T* births per year. Let *F*(*t*) and $\overset{Â¯}{F}(t)$ denote the matrices of coefficients (model parameters) in the differential equations for HIV-negative and HIV-positive populations, respectively. Let e_1_denote the 19-vector with a 1 in the first element and zeros elsewhere. We can now compactly denote eqs.(1) as:

(16) $$\frac{\text{d}x}{\text{d}t}=F(t)x+{\text{e}}_{1}T$$

Let *γ*denote the HIV infection rate, per person per year. Following M-S, we will move individuals from each HIV-negative category to the corresponding HIV-positive category at rate *γ*. Thus, instead of eq.(16), we have the following coupled sets of equations:

(17) $$\frac{\text{d}x}{\text{d}t}=F(t)x+{\text{e}}_{1}T-\mathrm{\gamma x}$$

(18) $$\frac{\text{d}\overset{Â¯}{x}}{\text{d}t}=\overset{Â¯}{F}(t)x+{\text{e}}_{1}\overset{Â¯}{T}+\mathrm{\gamma x}$$

The term ‘−*γx*’ in eq.(17) removes individuals from the HIV-negative population at the HIV infection rate, and the term ‘ + *γx*’ in eq.(18) introduces them into the HIV-positive population at the same rate.

M-S introduce further highly structured coupling between eqs.(17) and (18) through the TB infection rate, [18], p.23, *λ*. We do not employ the M-S differentiation between the infection rates for HIV-negative and HIV-positive populations. Instead we simply use *λ*and $\overset{Â¯}{\lambda }$ for the TB infection rates in the HIV-negative and HIV-positive populations.

### Uncertainty

Many uncertainties accompany the dynamic model. We concentrate on uncertainty in the values of some of the model parameters, as this is the dominant impact of HIV prevalence. We use info-gap theory to model and manage these uncertainties [8]. Many different types of info-gap models of uncertainty are available. We employ a model particularly suited to severe lack of information.

The dominant uncertain parameters are: 

$\rho_{S},{\overset{Â¯}{\rho }}_{S}$, slow relapse rates.

$\rho_{F},{\overset{Â¯}{\rho }}_{F}$, fast relapse rates.

$\lambda ,\overset{Â¯}{\lambda }$, TB infection rates.

*γ*, HIV infection rate.

Let us denote uncertain variables generically as *u*_*i*_, compiled in a vector *u*. This vector is:

(19) $$u=(\rho_{S},\rho_{F},\lambda ,{\overset{Â¯}{\rho }}_{S},{\overset{Â¯}{\rho }}_{F},\overset{Â¯}{\lambda },\gamma )$$

For each uncertain parameter, *u*_*i*_, we have an estimated value, denoted ${\tilde{u}}_{i}$, and an error term *s*_*i*_typically chosen as half of an interval estimate of the parameter. The error estimate may be derived from a statistical confidence interval, or from a plausible extension of a confidence interval as discussed by Grassly *et al*[32], or from other professional judgment. The basic idea of an info-gap model of uncertainty is that we don’t know how wrong our estimate is; we have no reliable estimate of a worst case. In fact, since the typical values are poorly known, worst-case estimates are even less reliable.

More precisely, the fractional error of the estimate, ${\tilde{u}}_{i}$, in units of the error, *s*_*i*_, is unknown. That is, this fractional error is bounded by a number, *α*, whose value is unknown:

(20) $$\left|\frac{u_{i}-{\tilde{u}}_{i}}{s_{i}}\right|\leq \alpha ,\;\alpha \geq 0$$

But this must be further refined to reflect the fact that the uncertain parameters are 1st-order removal-rate constants^b^, which means that they cannot be negative. Thus we adjoin these constraints to the inequality as:

(21) $$u_{i}>0,\;\left|\frac{u_{i}-{\tilde{u}}_{i}}{s_{i}}\right|\leq \alpha ,\;\alpha \geq 0$$

Finally, we write our info-gap model of uncertainty as a family of nested sets of uncertain vectors:

(22) $$U(\alpha )=\left\{u:\;u_{i}>0,\;\left|\frac{u_{i}-{\tilde{u}}_{i}}{s_{i}}\right|\leq \alpha ,\;\text{for all}\;i\right\},\;\alpha \geq 0$$

*α* is called the ‘horizon of uncertainty’. When *α*=0 there is no uncertainty and the set U(0) contains only the estimated values, $\tilde{u}$. As *α* increases, the sets U(α) become more inclusive. These sets are unbounded in the space on which the parameters are defined. The info-gap model embodies the information we have—estimates and errors—without committing to any meaningful worst case (other than the limits which are imposed by the definition of the variables).

In some situations one may not be able to estimate error weights, *s*_*i*_. In such situations the fractional error in eq.(20) can be replaced by a fractional error relative to the estimate, $\left|(u_{i}-{\tilde{u}}_{i})/{\tilde{u}}_{i}\right|$. The info-gap model is then formulated as in eq.(22) with this new fractional error.

### Robustness: formulation

#### Performance requirements

We will consider an aggregated variable for monitoring the TB status of the population. Our goal is to keep the value of this variable acceptably small. The variable we consider is the total number of cases, untreated and treated, HIV-positive and HIV-negative, as a fraction of the initial population:

(23) $$C(t)=\underset{i,j}{\sum }\left[C_{U}^{i,j}(t)+C_{T}^{i,j}(t)+{\overset{Â¯}{C}}_{U}^{i,j}(t)+{\overset{Â¯}{C}}_{T}^{i,j}(t)\right]$$

There are other variables that one could consider. For instance, one could consider the total number of relapses, fast and slow, HIV-positive and HIV-negative, as a fraction of the initial population:

(24) $$R(t)=R_{F}(t)+R_{S}(t)+{\overset{Â¯}{R}}_{F}(t)+{\overset{Â¯}{R}}_{S}(t)$$

One could also consider the instantaneous or the average rates of change of *C*(*t*) and *R*(*t*).

Returning to the aggregate prevalence, *C*(*t*), our goal is to keep it below a specified maximum acceptable value at a specified target time *t*_m_. Thus the performance requirement is:

(25) $$C(t_{\text{m}})\leq C_{\text{m}}$$

A relation such as eq.(25) is called a “satisficing” requirement, as opposed to an optimization requirement. We do not aim to minimize the aggregate prevalence, *C*(*t*_m_). Our goal is to make the TB prevalence adequately small: no greater than the critical value *C*_m_, as stated in eq.(25). Note that the satisficing requirement includes optimization as a special case. Satisficing and optimizing are the same when *C*_m_ is chosen as the predicted minimal value.

#### Control variables

We aim to achieve this goal by judicious choice of control variables that we denote generically as *q*_*i*_, combined in a vector *q*. Eligible control variables are any coefficients of the dynamic model that can be influenced by public health or related medical intervention. When a control variable is also an info-gap uncertain variable we will refer to the estimated value as the control variable. The uncertainty is then in whether the specified value—the estimate—will be realized in practice. We will consider the following control variables: 

$\delta^{j},{\overset{Â¯}{\delta }}^{j}$, diagnosis rates (same for HIV negative and positive populations).

$\epsilon_{T}^{k},{\overset{Â¯}{\epsilon }}_{T}^{k}$, cure rates for treateds (same for HIV negative and positive populations).

${\tilde{\rho }}_{F},{\tilde{\overset{Â¯}{\rho }}}_{F}$, estimated fast relapse rates.

$\tilde{\lambda },\tilde{\overset{Â¯}{\lambda }}$, estimated TB infection rates.

$\tilde{\gamma }$, estimated HIV infection rate.

$\beta_{F},{\overset{Â¯}{\beta }}_{F}$, fast breakdown rates.

We define an intervention in terms of the values of these variables. None of these control variables corresponds directly to any of the standard performance measures such as the incidence, prevalence, and death rates associated with TB. For instance, the coefficients *δ*^*j*^and ${\overset{Â¯}{\delta }}^{j}$, while called “diagnosis rates”, are in fact 1st-order kinetic rate coefficients and can meaningfully take any positive value. These coefficients combine with several other coefficients to determine the fraction of new untreated cases that move into the treated category, as seen from eqs.(6) and (7). In other words, the control variables combine to produce aggregate effects such as the proportion of new cases that are diagnosed. One can “calibrate” a set of control variables in terms of aggregate properties, for instance by keeping track of how many cases are created (new members of *C*_*U*_(*t*)) and how many are treated (new members of *C*_*T*_(*t*)). Unless the population is at steady state (and the intervention tries to prevent this), the calibration in terms of the proportion diagnosed depends on the time after initiation of intervention and on the duration during which the accounting is done. We do not calibrate our model since we focus on a different challenging problem: prioritizing alternative interventions.

#### Definition of robustness

An intervention is specified by specifying the values of the control variables, *q*. If our dynamic model were accurate we could evaluate any proposed intervention in terms of the outcome of that intervention that is predicted by the model. An intervention whose predicted outcome entails low TB prevalence is preferred over an intervention with larger predicted prevalence.

The problem is that the dynamic model is highly uncertain. This means that it is unrealistic to prioritize interventions in terms of their predicted outcomes. Since those predictions are highly uncertain, it is unwise to evaluate interventions only in terms of their model-based predictions.

The model-based predictions are useful, but we also ask: how wrong could the model be, and the predicted outcome is still acceptable? That is, for any specified intervention, *q*, we ask: what is the largest fractional error in the uncertain parameters, up to which all realizations of the model would yield acceptable outcomes? The answer to that question is the robustness function, which we will soon specify. We use the robustness function to prioritize the interventions in terms of their robustness against uncertainty for achieving the required outcomes.

The robustness function for the performance requirement in eq.(25) is:

(26) $$\hat{\alpha }(q,C_{\text{m}})=\max\left\{\alpha :\;\left(\underset{u\in U(\alpha )}{\max}C(t_{\text{m}},u)\right)\leq C_{\text{m}}\right\}$$

We can “read” this relation from left to right as follows. The robustness, $\hat{\alpha }$, of intervention *q*, with performance requirement *C*_m_, is the maximum horizon of uncertainty, *α*, up to which the maximum aggregate prevalence, *C*(*t*), for all realizations of the uncertain coefficients *u* in the info-gap model U(α), does not exceed the critical value, *C*_m_. We are not ameliorating a worst case; the worst case is unknown because the horizon of uncertainty, *α*, is unbounded. Instead, we are asking how large an uncertainty can be tolerated by the intervention, *q*. In choosing the intervention to enhance the robustness, we attempt to protect against the unbounded uncertainty of the impact of HIV/AIDS on the TB dynamics.

## Endnotes

^a^Footnote 1 in the full online version, pp. 20–21.^b^This means that these parameters are the coefficients in equations such as $\dot{x}(t)=-\mathrm{ux}(t)$ whose solution is *x*(*t*)=*x*(0)e^−*ut*^. In order for this to be a removal process, the coefficient *u* must be positive. It can exceed unity.

## Abbreviations

AIDS: Acquired immunodeficiency syndrome; HIV: Human immunodeficiency virus; RDM: Robust Decision Making.

## Competing interests

The authors have no competing interests.

## Authors’ contributions

YB-H formulated the decision analysis and implemented the calculations. CD and NZ formulated the medical model. All authors had access to all data, participated in interpreting the results of the analysis, contributed to writing the manuscript and approved the last version of the manuscript.

## Pre-publication history

The pre-publication history for this paper can be accessed here:

http://www.biomedcentral.com/1471-2458/12/1091/prepub
